# Associations between pain sensitization and measures of physical function in people with hand osteoarthritis: Results from the Nor-Hand study

**DOI:** 10.1016/j.joca.2023.07.005

**Published:** 2023-07-24

**Authors:** Marthe Gløersen, Pernille Steen Pettersen, Tuhina Neogi, Joseph Sexton, Tore K. Kvien, Hilde Berner Hammer, Ida K. Haugen

**Affiliations:** § Center for Treatment of Rheumatic and Musculoskeletal Diseases (REMEDY), Diakonhjemmet Hospital, Oslo, Norway; † University of Oslo, Faculty of Medicine, Oslo, Norway; ‡ Section of Rheumatology, Boston University School of Medicine, Boston, United States of America

**Keywords:** Osteoarthritis, Hand osteoarthritis, Pain, Function, Pain sensitization

## Abstract

**Objective:**

To examine whether pain sensitization is associated with hand and lower extremity function in people with hand osteoarthritis (OA) in the Nor-Hand study.

**Design:**

Pain sensitization was assessed by pressure pain thresholds (PPTs) and temporal summation (TS). Hand function was assessed by Australian/Canadian Osteoarthritis Hand Index (AUSCAN) (range: 0–36), grip strength and Moberg pick-up test, and lower extremity function was assessed by Western Ontario and McMaster Universities Osteoarthritis Index (range: 0–68), 30-s chair stand test, and 40-m walk test. We examined whether sex-standardized PPT and TS values were cross-sectionally associated with measures of physical function using linear regression analyses. Beta coefficients were presented per sex-specific standard deviation of PPT and TS. The mediating effect of pain was examined by causal-inference based mediation analysis.

**Results:**

In 206 participants, higher PPTs at/near the hand, indicative of less peripheral and/or central pain sensitization, were associated with greater grip strength and better self-reported hand function (beta for PPT at finger joint on AUSCAN function: −1.41, 95% CI −2.40, −0.42). Higher PPTs at/near the hand, near the knee and at trapezius were associated with lower extremity function, although not statistically significant for all outcomes. Self-reported pain severity mediated the effect of PPT on self-reported function. TS was not associated with hand or lower extremity function.

**Conclusion:**

Peripheral sensitization, and possibly central sensitization, was associated with impaired function. Effects of PPTs on self-reported function were mediated by self-reported pain, whereas there might be a direct effect of sensitization or effects through other mediators on performance-based function.

## Introduction

Hand osteoarthritis (OA) causes substantial disability and suffering, and pain relief is usually limited with currently available treatment. Pain is the primary symptom of OA, often accompanied by impaired physical function.^1^ OA in the lower extremities is a leading cause of functional limitations among elderly people,^2^ mainly because of pain. However, the specific mechanisms for how pain may affect physical function is not known. Worsening of knee pain predicts deterioration of physical functioning in people with knee OA,^3^ and low physical functioning is 50% more common in people with bilateral knee pain compared with people with unilateral pain.^4^ Elderly people with symptomatic hand OA have weaker grip strength, and more difficulty in carrying a bundle, handling small objects and writing compared with elderly people without symptomatic hand OA.^5^ Hand pain is also associated with self-reported functional limitations and reduced grip strength in people with hand OA.^6-8^

In recent years, research has focused on understanding the neurobiological mechanisms involved in OA-related pain, including the role of pain sensitization;^9,10^ Neuroplastic changes in the peripheral and central nervous system such as pain sensitization may manifest as local and widespread hyperalgesia, allodynia, enlargement of receptive fields and chronicity of pain.^9^ Greater pain sensitization is associated with higher pain severity in people with knee and hand OA,^11,12^ and people with OA often have greater pain sensitization than healthy controls.^10^ Some previous knee OA studies have also suggested that pain sensitization is related to altered motor activity and functional limitations,^13-21^ while there is limited evidence for such an association in a hand OA population.^6,22,23^ A recent study of persons with or at risk of knee OA showed that pain severity mediated the relation between pain sensitization and self-reported function, whereas pain severity did not seem to meaningfully mediate the relation between sensitization and objective measures of function.^21^ No previous studies have assessed whether central sensitization affects both upper and lower extremity function, although central sensitization can affect all parts of the body.

Several studies have found that neuromuscular adaptations occur in response to pain, but the specific mechanisms and extent of these adaptations are debated.^24^ The neuromuscular adaptations may involve a reorganization of the motor control system with increased or decreased muscle activity, which may lead to disability and functional impairment.^24^ However, the extent to which pain sensitization may impact functioning in hand OA is not known. Hence, we assessed whether pain sensitization was associated with impaired hand and lower extremity function in people with hand OA, and whether this relation was mediated by self-reported pain severity.

## Methods

### Study design and population

We used cross-sectional data from the follow-up examination (2019–2021) of the hospital-based Nor-Hand study.^25^ Follow-up data were used due to a broader collection of measures of physical function in this data collection and better reliability of the quantitative sensory testing (QST), based on intraclass correlation coefficients (ICCs), than at baseline. The clinical examination of the follow-up study included participants with hand OA diagnosed by a rheumatologist with ultrasound and/or clinical examination. All participants were between 40 and 70 years of age at the baseline visit (2016–2017) and were recruited through the outpatient clinic or a one-day multidisciplinary OA course at Diakonhjemmet Hospital (Oslo, Norway). The most important exclusion criteria were systemic inflammatory joint diseases, hemochromatosis, and psoriasis. The detailed inclusion and exclusion criteria have been described in the protocols from the baseline and follow-up examination.^25,26^ The study was approved by the Norwegian Regional Committee for Medical and Health Research Ethics (Ref. no: 2019/363) and by the data protection officer at Diakonhjemmet Hospital. The procedures followed were in accordance with the Helsinki Declaration. Written consent was obtained from all participants after they had received oral and written information about the study. The study is registered at https://clinicaltrials.gov (Ref.no: NCT03083548).

### Quantitative sensory testing

Pressure pain thresholds (PPT) were tested at one interphalangeal finger joint (selection described below), the left distal radioulnar joint, the trapezius muscle, and the tibialis anterior muscle. The interphalangeal finger joint evaluated at this follow-up visit had originally been selected at the baseline visit as the most painful interphalangeal joint, or alternatively the joint with the most severe clinical OA with swelling and/or bony enlargements if no joints were painful. The same finger joint was tested at the follow-up visit, and we asked the participants whether the tested finger joint was still painful. A digital hand-held algometer (FPIX25, Wagner instruments, or FPIX50 if we measured values > 10 kg/cm^2^) was placed perpendicular to the joint/muscle and the pressure was increased at a rate of 0.5 kg per second guided by a metronome. The PPT was defined as the point at which the participant first indicated that the application of pressure became slightly painful. The average of three measurements at each site taken with 30 s intervals in between was used in the analyses at the PPT for the given anatomic site.

A set of seven weighted probes (8, 16, 32, 64, 128, 256 and 512 mN) was used to measure temporal summation (TS). First, the lightest probe (8 mN) was tapped against the left distal radioulnar joint once, and the participants rated the pain intensity on a Numerical Rating Scale (NRS, ranging from 0 = no pain to 10 = worst pain imaginable). All probes were then tapped consecutively with increasing weight until the participant rated the pain as ≥ 4 on the NRS. The probe that caused a pain rating ≥ 4, or alternatively the heaviest probe if the participant did not report a pain intensity ≥ 4 with the highest weight probe, was used for further testing. This probe was tapped 10 times (one tap per second) against the radioulnar joint. Pain intensity of the first, fifth and tenth tap was rated on the NRS. The procedure was repeated after three minutes. TS was calculated as the pain rating of the first tap subtracted from the maximum pain rating of the fifth and tenth taps. The average of the two TS measurements was used in the analyses.

QST was performed by a trained physician (MG) and a medical student. Eleven participants were examined by both examiners on the same afternoon. The inter-assessor reliability was good to very good, with ICCs (two-way mixed-effects model, absolute agreement, individual measure) of 0.83 for PPT at the interphalangeal finger joint, 0.73 for PPT at the radioulnar joint, 0.75 for PPT at the trapezius muscle, 0.85 for PPT at the tibialis anterior muscle and 0.84 for TS. The ICC values were interpreted similar to kappa values.^27^

### Questionnaires

Participants answered the Australian/Canadian Osteoarthritis Hand Index (AUSCAN) pain (range: 0–20) and physical function (range: 0–36) subscales and the Western Ontario and McMaster Universities Osteoarthritis Index (WOMAC) pain (range: 0–20) and physical function (range: 0–68) subscales concerning their hand and knee/hip symptoms, respectively, during the last 48 h.^28-30^

### Performance-based measures of physical function

All participants tested their grip strength with the Jamar dynamometer supervised by a trained medical student.^31^ They were instructed to squeeze the dynamometer as hard as possible, whilst sitting in a chair with a neutral shoulder position, without any support under the arms and a 90° angle in the elbows. Both hands were tested three times with 15 s of rest between the assessments, and the average grip strength (in kilograms) was calculated in each hand. NRS pain (range: 0–10) in each hand immediately after the three grip strength measurements was assessed.

The Moberg pick-up test was used to investigate the fine motors skills of both hands.^32^ The participants were sitting in front of a table with twelve metallic objects spread on a white paper within a radius of 20–30 cm. Participants were asked to pick up the objects one-by-one as fast as they could and put them in a small box. Time in seconds (with one decimal) used to pick up all the twelve objects was recorded for each hand.

The 30-s chair stand test was used to assess lower extremity function.^33,34^ A chair without armrest and the same height of the seat (44 cm) was used throughout the study. Participants were instructed to sit with their feet shoulder-width apart and with their arms crossed close to the chest. If the participants wore inappropriate footwear, they performed the test in bare feet. The participants were instructed to stand completely up and sit back down for as many times as possible within 30 s. The number of chair stands (up and down) was counted. The final stand was included in the count if the allotted time expired when the participant was standing. If the participant could not perform one single stand without using their arms or an assistive device, the score for the test was 0.

The 40-m fast-paced walk test was performed on a ten meter-walkway marked in each end with tape and cones.^33,34^ The test was performed without shoes if the participants wore inappropriate footwear. Participants were instructed to walk 40 m (4 * 10 m; 3 turns) as quickly as possible, without running. Time in seconds (two decimals) was recorded. Walking aids were allowed, and it was recorded if a walking aid was utilized. One person was not able to perform the test and was not included in the analyses. The examiner followed the participants throughout the test if needed to ensure safety.

### Potential confounders

Potential confounders included age, sex, body mass index (BMI), radiographic/ultrasound-detected severity of OA, education, and physical activity. At the follow-up visit, height and weight were measured in light indoor clothing and without shoes, and BMI (kg/m^2^) was calculated. Because we wanted to adjust our analyses for OA severity, a modified Kellgren-Lawrence grade was used by a trained physician (IKH) to determine radiographic hand OA severity in 32 joints (range: 0–128).^35^ An ultrasound assessment of the lower extremities was performed by two trained physicians. Ultrasound-detected osteophytes were scored on 0–3 scales, and a sum score of osteophytes in the bilateral knees (maximum score of four investigated compartments in each knee), hips, and feet (first metatarsophalangeal joints) (range: 0–18) was calculated. Six participants were examined by both physicians on the same afternoon to assess the inter-assessor reliability, which was found to be very good with an ICC (two-way mixed-effects model, absolute agreement, individual measure) of 0.88 for the calculated sum score.^27^ The highest degree of completed education (7 levels) was collected at baseline and was not repeated at this follow-up visit. Physical activity was assessed at this follow-up visit with three questions about frequency, intensity, and duration. A previously validated sum score of weekly physical activity weighted by intensity level (range: 0–15) was calculated as the product of the frequency, intensity and duration scales.^36^ Higher values indicate greater levels of physical activity. The participants were also asked about handedness at the follow-up visit.

### Statistical analyses

We created sex-standardized PPT and TS values by subtracting the sex-specific mean from the observed value of each participant and dividing by the sex-specific standard deviation (SD). The sex-standardized values were calculated for men and women separately because of sex differences in pain sensitivity.^37^ These values are expressed as the number of SD from the average, e.g., a value of 0 in a man represents the average PPT/TS value in men, whereas a value of 1 represents one SD above the average in men, and correspondingly for women. We assessed associations between measures of pain sensitization and physical function using linear regression. Assumptions of linear regression analyses were assessed through graphical means, e.g., by scatter plots and histograms. In analyses of PPTs at the trapezius and tibialis anterior muscles and TS, grip strength and fine motor skills (Moberg test) in the dominant hand were used as outcomes. In analyses of PPTs at the finger joint and the radioulnar joint, we used grip strength and fine motor skills measured in the same hand as where the PPTs were tested. These analyses were additionally adjusted for handedness. All analyses were adjusted for age, sex, BMI, OA severity (i.e., Kellgren-Lawrence sum score of the hands in the analyses of hand function and a sum score of ultrasound-detected osteophytes in the hips, knees, and feet in the analyses of lower extremity function), education and physical activity.

Pain severity was not included as a potential confounder in the main analyses because it may be an intermediate in the causal pathway between pain sensitization and physical function. Instead, we performed a causal-inference based mediation analysis by fitting natural-effects models in order to estimate the natural indirect effect of pain sensitization on physical function potentially mediated through pain severity (AUSCAN hand pain in the analyses with AUSCAN function and the Moberg pick-up test as outcomes, NRS pain after grip strength measurements in the analyses with grip strength as outcome and WOMAC knee/hip pain in the analyses with outcome measures of lower extremity function), and the natural direct effect of pain sensitization on physical function not mediated through pain severity. This estimation is based on the counterfactual framework for causal inference.^38^ We used the imputation-based approach for mediation analyses.^39^ Estimates for total, natural direct and natural indirect effects are presented per sex-specific SD of PPT and TS with 95% confidence intervals (CI) estimated by bootstrapping.

Multiple imputation was used to deal with missing values using 50 imputations from a multivariate normal model. The main analyses were performed using Stata/IC version 16, and the mediation analyses were performed using the medflex package in the R software version 4.0.2. P-values less than 0.05 were considered statistically significant.

## Results

### Participant characteristics

Among the 300 participants who were enrolled in the Nor-Hand cohort in 2016–2017, 213 participants attended the follow-up visit in 2019–2021. In our analyses, we included 206 participants (Table 1) after exclusion of 6 participants with missing clinical measures. The median (interquartile range) age was 65 (60–69) years and 86% were women. A quarter (25%) of the participants took pain medications (oral or topical nonsteroidal anti-inflammatory drugs, acetaminophen or opioids/opioid-like drugs) on the same day they underwent QST. The mean grip strength in the dominant hand was 21.2 kg, participants completed on average 12.1 chair stands in 30 s and had a mean walking speed of 1.8 meters/second (m/s). One person used a walking aid to perform the walk test.

### Sensitization and measures of physical function

Only 75 (36.4%) of the participants indicated that they had pain at the follow-up visit in the interphalangeal finger joint that was used as a test site for PPT because it was painful or had the most severe clinical OA at the baseline visit. In general, higher PPTs (indicating less sensitization) were associated with better function in hands and lower extremities, although not statistically significant for all outcomes (Tables 2 and 3). For hand function, the associations were stronger for PPTs measured at or near the hand (the finger joint and the radioulnar joint, as measures of peripheral and/or central sensitization) than for PPTs measured at distant sites (as measures of central sensitization). There was a trend towards an association between PPTs at the finger joint and the Moberg pick-up test (Table 2).

Higher PPTs were associated with better self-reported WOMAC lower extremity function and a higher walking speed (Table 3). In contrast to the results on hand function, the strength of associations was similar across the four PPTs, including sites close to the knee (tibialis anterior muscle), which may be a measure of peripheral and/or central sensitization in people with knee OA, and distant sites. The associations were not statistically significant for the chair stand test, although the associations were in the same direction as WOMAC function and the 40-m walk test (Table 3). No statistically significant associations were found between TS and hand or lower extremity function (Tables 2 and 3).

### Mediation analyses

The causal-inference based mediation analyses suggested that the effects of PPTs on physical function were partially mediated by self-reported pain severity. Effect sizes for mediation by pain severity were larger for self-reported function (AUSCAN hand function and WOMAC knee/hip function) than for performance-based funcion (grip strength and walking speed) (Online Supplementary Tables 1 and 2). As an example, the indirect effect of PPT at the radioulnar joint on AUSCAN function mediated through hand pain was −1.18 (95% CI: −1.95, −0.41), whereas the direct effect was −0.20 (95% CI: −0.89, 0.49). In contrast, the indirect effect of PPT at the radioulnar joint on grip strength mediated through NRS hand pain was 0.12 (95% CI: −0.10, 0.34) and the direct effect was 1.20 (95% CI: 0.27, 2.13) (Online Supplementary Table 1). Similar trends were seen in the analyses of lower extremity function, where for example the indirect effect of PPT at the trapezius muscle on WOMAC function mediated by WOMAC knee/hip pain was −1.59 (95% CI: −2.93, −0.26) and the direct effect was −0.14 (95% CI: −1.27, 1.00). The indirect effect of PPT at the trapezius muscle on walking speed mediated by knee/hip pain was 0.01 (95% CI: −0.00, 0.02) and the direct effect was 0.02 (95% CI: −0.01, 0.06) (Online Supplementary Table 2).

## Discussion

This is the first study to comprehensively explore associations between pain sensitization and measures of both hand and lower extremity function in people with hand OA. We found that higher PPTs measured at or near the hand or knee (indicative of less peripheral and/or central pain sensitization) were associated with better self-reported and performance-based hand and lower extremity function. PPTs at the trapezius muscle (indicative of central sensitization) were associated with lower extremity function, whereas no associations were found between TS (measure of central sensitization) and hand or lower extremity function. Pain severity partially mediated the effects of PPTs on physical function. Effect sizes for mediation by pain severity were larger for self-reported hand and lower extremity function than for grip strength and walking speed. Investigating whether pain sensitization is associated with physical function is relevant since targeting sensitization might not only reduce pain, but also functional decline in people with hand OA.

To our knowledge, only a few small hand OA studies have previously investigated the relationship between pain sensitization and hand function.^6,22,23^ Sofat et al. found that PPTs at the hands correlated with AUSCAN function.^23^ However, few correlations were found between PPTs or other QST parameters and upper extremity function in two other hand OA studies.^6,22^ In patients with shoulder OA, lower preoperative electrical pain threshold on the affected side, but not on the contralateral side, was associated with impaired self-reported arm, shoulder, and hand function 12 months after total shoulder arthroplasty.^40^ In line with the results from this study, we found that PPTs measured at or near the hand, as a measure of peripheral and/or central pain sensitization, were associated with self-reported AUSCAN hand function. Higher PPTs measured at or near the hand were also associated with greater grip strength. Few studies have assessed the minimal clinically important improvement (MCII) or the minimal clinically important difference (MCID) for grip strength and AUSCAN function in people with hand OA. However, a MCII for AUSCAN function of 3.3 (0–36 scale),^41^ and a MCID for grip strength of 1 kg have been proposed.^42^ Based on these estimates, our results may be clinically relevant because two persons would need to have a difference of approximately 1 SD in PPTs at the finger joint and the radioulnar joint to have a clinically important difference in grip strength. However, a larger difference in PPTs is needed to have a clinically important difference in AUSCAN function according to our study.

Previous studies have found relationships between lower PPTs, measured at both local and distant sites, and self-reported and measured functional limitations and altered motor activity among knee OA patients.^13-15,17-21^ However, two small studies of persons with knee and hip OA did not find any correlations between PPTs and function,^43,44^ and most of the QST variables assessed pre-surgically in a recent study were not predictive of WOMAC function six months after total knee replacement ^45^ Although we found associations between PPTs and measures of lower extremity function, these associations were not statistically significant for all outcomes. A statistically significant association with walking speed was found for PPT at the tibialis anterior muscle, which is close to the knee joint and therefore may not only represent central sensitization, but also peripheral sensitization in the participants that have concomitant knee OA (42% of our study population). The MCII of WOMAC function has been estimated to be 4.1 on a 0–68 scale,^46^, and a difference of 0.2–0.3 m/s in the 40-m walk test is considered to be clinically important in patients with hip OA.^47^ Thus, the associations between PPTs and measures of lower extremity function observed in our study seem to be of limited clinical relevance.

Causal inference-based mediation analyses suggested that the effects of PPTs on hand and lower extremity function were partially mediated by pain severity. Effect sizes for mediation by pain severity were larger for self-reported function than for measures of performance-based function like grip strength and walking speed. This inconsistency may be explained by a strong correlation between self-reported measures of pain and function, while the associations between pain and physical performance tests are less pronounced. Similar results were found in the Multicenter Osteoarthritis (MOST) study of persons with or at risk of knee OA, where the greatest mediation by self-reported pain was seen in the analyses of self-reported lower extremity function and only minimal mediation in the analyses of performance-based function.^21^

TS was not associated with any of the measures of physical function in our study. Our findings are in contrast with results from the MOST study, where presence of TS was associated with greater hamstring co-contraction during a knee extension task, with worse self-reported WOMAC function and with slower walking speed.^13,21^ The authors suggested that these findings may be due to shared central mechanisms of motor function and pain sensitization. Further, a recent study found that TS measured at the knee, but not extra-segmentally at the forearm, was associated with the 40-m walk test and the stair climb test among persons with painful knee OA. However, no association with the 30-s chair stand test was found.^16^

There are some limitations to our study. We used cross-sectional data, and the possibility of reverse causation can consequently not be excluded. We adjusted for several possible confounders, but residual confounding by unmeasured confounders might have biased our results. Some of the results showed trends without being statistically significant, which may be because of lack of precision due to the restricted sample size (n = 206). Participants were recruited from secondary care, which may impact the generalizability of our results. We did not instruct participants to hold pain medications on the day they were examined, but use of pain medications was recorded. Most of the participants performed well on the chair stand test (mean: 12.1 repetitions) and walk test (mean walking speed: 1.8 m/s) compared with normative data,^34^ whereas we have previously shown that their grip strength was lower than in the general population at baseline.^7^ With a larger variation especially in the performance-based tests of lower extremity function, we might have found stronger associations in our study. We asked about hand pain right after, and not during, grip strength measurements, which may have underestimated the degree of pain.

In summary, our findings suggest that higher PPTs at or near the hands or knees, that may mainly represent peripheral sensitization, are associated with better hand and lower extremity function. In addition, PPT at the trapezius muscle, but not TS, as measures of central sensitization, was associated with lower extremity function. Mediation analyses suggested that the effects of pain sensitization on self-reported function were mediated by self-reported pain, whereas pain sensitization may have direct effects or there may be effects through other mediators on grip strength and walking speed.

## Supplementary Material

Supplement

Supplement 2

## Figures and Tables

**Table 1:** Demographic and clinical characteristics of the 206 participants.

Age, median (IQR) years	65 (60–69)
Women, n (%)	178 (86)
Fulfillment of ACR hand OA criteria, n (%)	171 (83)
Fulfillment of ACR knee OA criteria, n (%)^a^	85 (42)
Body mass index, mean (SD) kg/m^2a^	27.3 (4.9)
Kellgren-Lawrence sum score (0–128), median (IQR)^a^	31 (18–48)
Sum score of ultrasound-detected osteophytes in lower extremities (0–18), median (IQR)	4 (2–6)
Educational level, n (%) with university or other higher Education^a^	124 (60)
Physical activity sum score (0–15), median (IQR)^a^	4 (2–5)
AUSCAN hand pain subscale (0–20), median (IQR)^a^	7 (5–10)
AUSCAN hand function subscale (0–36), median (IQR)^a^	12 (6–18)
WOMAC knee/hip pain subscale (0–20), median (IQR)^a^	4 (1–7)
WOMAC knee/hip function subscale (0–68), median (IQR)^a^	10 (2–23)
Grip strength dominant hand, mean (SD) kg^a^	21.2 (9.1)
Pain in dominant hand after grip strength measurements (0–10), median (IQR)^a^	2 (0–4)
Moberg pick-up test dominant hand, median (IQR) seconds	14.1 (12.5–16.9)
Walking speed, mean (SD) meters/second^a^	1.8 (0.3)
Chair stand test, mean (SD) number of complete stands in 30 s^a^	12.1 (3.7)
PPT finger joint, mean (SD) kg/cm^2b^	3.6 (1.6)
PPT radioulnar joint, mean (SD) kg/cm^2^	4.1 (2.0)
PPT trapezius muscle, mean (SD) kg/cm^2a^	4.1 (2.2)
PPT tibialis anterior muscle, mean (SD) kg/cm^2^	4.8 (2.4)
Temporal summation, median (IQR)	1.5 (0.5–2.5)

IQR, interquartile range; ACR, American College of Rheumatology; SD, standard deviation; PPT, pressure pain threshold; MTP, metatarsophalangeal.

a Missing values: Fulfillment of ACR knee OA criteria n = 2, BMI n = 5, Kellgren-Lawrence sum score n = 5, educational level n = 1, physical activity n = 4, AUSCAN hand pain n = 3, AUSCAN hand function n = 1, WOMAC knee/hip pain n = 5, WOMAC knee/hip function n = 2, grip strength dominant hand n = 1, pain after grip strength measurements in dominant hand n = 1, walking speed n = 1, chair stand test n = 1 (one observation was excluded from the analyses because the participant did not sit completely back down on the chair on all repetitions), PPT trapezius n = 1.

b n = 75 (36.4%) had pain in the finger joint that was used as a test site for PPT.

**Table 2:** Associations between measures of pain sensitization and hand function.

	AUSCAN function (range 0–36)	Grip strength (kilograms)^a^	Moberg pick-up test (seconds)^a^
	Beta (95% CI)	Beta (95% CI)	Beta (95% CI)
PPT finger joint	**−1.41** **(−2.40, −0.42)**	**1.23** **(0.33, 2.13)**	−0.67 (−1.43, 0.10)
PPT radioulnar joint	**−1.38** **(−2.37, −0.38)**	**1.32** **(0.40, 2.23)**	−0.13 (−0.82, 0.55)
PPT trapezius muscle	−0.80 (−1.82, 0.21)	0.66 (−0.25, 1.57)	−0.27 (−1.01, 0.46)
PPT tibialis anterior muscle	−0.76 (−1.77, 0.25)	0.74 (−0.17, 1.64)	−0.30 (−1.04, 0.43)
Temporal summation	−0.07 (−1.06, 0.92)	−0.28 (−1.17, 0.60)	0.12 (−0.59, 0.84)

Adjusted for: age, sex, body mass index, Kellgren-Lawrence sum score of the hands, education and physical activity.

Presented per sex-specific standard deviation (SD) of the PPT and TS values (SD for PPT at the finger joint in women=1.54 kg/cm^2^ and men=1.78 kg/cm^2^, SD for PPT at the radioulnar joint in women=1.60 kg/cm^2^ and men=2.93 kg/cm^2^, SD for PPT at trapezius in women=1.73 kg/cm^2^ and men=3.72 kg/cm^2^, SD for PPT at tibialis anterior in women=1.90 kg/cm^2^ and men=3.62 kg/cm^2^, SD for temporal summation in women=1.74 and mem=1.24).

AUSCAN, Australian/Canadian Osteoarthritis Hand Index; PPT, pressure pain threshold.

a Grip strength and Moberg test are measured in the same hand as PPT in the analyses of PPT at the finger joint and radioulnar joint (additionally adjusted for handedness), and in the dominant hand in the analyses of the other measures of pain sensitization.

**Table 3:** Associations between measures of pain sensitization and lower extremity function.

	WOMAC function (range 0–68)	Chair stand test (number of stands)	40-m walking speed (m/s)
	Beta (95% CI)	Beta (95% CI)	Beta (95% CI)
PPT finger joint	**−1.73** **(−3.41, −0.05)**	0.25 (−0.22, 0.71)	0.03 (−0.01, 0.06)
PPT radioulnar joint	−1.65 (−3.37, 0.06)	0.25 (−0.23, 0.73)	0.03 (−0.01, 0.06)
PPT trapezius muscle	**−1.73** **(−3.45, −0.01)**	0.19 (−0.29, 0.68)	**0.04** **(0.00, 0.07)**
PPT tibialis anterior muscle	−1.58 (−3.30, 0.14)	0.12 (−0.36, 0.60)	**0.04** **(0.01, 0.08)**
Temporal summation	0.93 (−0.78, 2.65)	−0.08 (−0.56, 0.40)	−0.01 (−0.04, 0.03)

Adjusted for: age, sex, body mass index, ultrasound-detected osteophytes in the knees, hips and feet, education and physical activity.

Presented per sex-specific standard deviation (SD) of the PPT and TS values (SD for PPT at the finger joint in women=1.54 kg/cm^2^ and mem=1.78 kg/cm^2^, SD for PPT at the radioulnar joint in women=1.60 kg/cm^2^ and mem=2.93 kg/cm^2^, SD for PPT at trapezius in women=1.73 kg/cm^2^ and men=3.72 kg/cm^2^, SD for PPT at tibialis anterior in women=1.90 kg/cm^2^ and men=3.62 kg/cm^2^, SD for temporal summation in women=1.74 and men=1.24).

WOMAC, Western Ontario and McMaster Universities OA Index; m/s, meters per second; PPT, pressure pain threshold.
