# Supplementary material for: Associations between pain sensitization and measures of physical function in people with hand osteoarthritis: Results from the Nor-Hand study
Source: Osteoarthritis Cartilage. Author manuscript; Available in PMC 2024 Oct 1. (PMC10528207; doi:10.1016/j.joca.2023.07.005)
Supplement: Supplement [file NIHMS1930749-supplement-Supplement.docx]

**Online Supplementary Table 1. Estimates of the total effect of a standard deviation (SD) increase in measures of pain sensitization on hand function and the corresponding natural direct effects and natural indirect effects mediated by hand pain**

|  | AUSCAN function (range 0-36) | Grip strength (kilograms)* | Moberg pick-up test (seconds)* |
| --- | --- | --- | --- |
| **PPT finger joint** | | | |
| Total effect | **-1.41 (-2.48, -0.34)** | **1.23 (0.33, 2.13)** | **-0.67 (-1.28, -0.05)** |
| Direct effect | 0.02 (-0.70, 0.73) | **1.08 (0.15, 2.00)** | -0.43 (-1.00, 0.15) |
| Indirect effect | **-1.43 (-2.13, -0.73)** | 0.15 (-0.04, 0.34) | **-0.24 (-0.43, -0.04)** |
| **PPT radioulnar joint** | | | |
| Total effect | **-1.38 (-2.37, -0.39)** | **1.32 (0.40, 2.24)** | -0.13 (-0.68, 0.41) |
| Direct effect | -0.20 (-0.89, 0.49) | **1.20 (0.27, 2.13)** | -0.01 (-0.57, 0.56) |
| Indirect effect | **-1.18 (-1.95, -0.41)** | 0.12 (-0.10, 0.34) | -0.13 (-0.27, 0.02) |
| **PPT trapezius muscle** | | | |
| Total effect | -0.80 (-1.88, 0.28) | 0.66 (-0.25, 1.56) | -0.27 (-0.92, 0.37) |
| Direct effect | 0.30 (-0.45, 1.05) | 0.58 (-0.34, 1.49) | -0.04 (-0.67, 0.60) |
| Indirect effect | **-1.10 (-1.87, -0.33)** | 0.08 (-0.06, 0.23) | **-0.24 (-0.44, -0.04)** |
| **PPT tibialis anterior muscle** | | | |
| Total effect | -0.76 (-1.73, 0.21) | 0.74 (-0.34, 1.81) | -0.30 (-0.92, 0.31) |
| Direct effect | -0.15 (-0.93, 0.64) | 0.62 (-0.48, 1.73) | -0.17 (-0.76, 0.41) |
| Indirect effect | -0.61 (-1.30, 0.07) | 0.11 (-0.06, 0.28) | -0.13 (-0.30, 0.04) |
| **Temporal summation** | | | |
| Total effect | -0.07 (-1.02, 0.88) | -0.28 (-1.13, 0.56) | 0.12 (-0.54, 0.79) |
| Direct effect | -0.16 (-0.79, 0.47) | -0.22 (-1.07, 0.62) | 0.10 (-0.52, 0.73) |
| Indirect effect | 0.09 (-0.69, 0.88) | -0.06 (-0.20, 0.08) | 0.02 (-0.15, 0.19) |
| *Grip strength and Moberg test are measured in the same hand as PPT in the analyses of PPT at the finger joint and radioulnar joint (additionally adjusted for handedness), and in the dominant hand in the analyses of the other measures of pain sensitization.  Adjusted for: age, sex, body mass index, Kellgren-Lawrence sum score of the hands, education and physical activity.  The effect estimates represent the estimated average increase in physical function outcomes, with corresponding 95% confidence intervals estimated by bootstrapping, presented per sex-specific standard deviation (SD) of the PPT and TS values (SD for PPT at the finger joint in women=1.54 kg/cm^2^ and men=1.78 kg/cm^2^, SD for PPT at the radioulnar joint in women=1.60 kg/cm^2^ and men=2.93 kg/cm^2^, SD for PPT at trapezius in women=1.73 kg/cm^2^ and men=3.72 kg/cm^2^, SD for PPT at tibialis anterior in women=1.90 kg/cm^2^ and men=3.62 kg/cm^2^, SD for temporal summation in women=1.74 and men=1.24).  AUSCAN, Australian/Canadian Osteoarthritis Hand Index; PPT, pressure pain threshold | | | |
