# Supplementary material for: Associations between pain sensitization and measures of physical function in people with hand osteoarthritis: Results from the Nor-Hand study
Source: Osteoarthritis Cartilage. Author manuscript; Available in PMC 2024 Oct 1. (PMC10528207; doi:10.1016/j.joca.2023.07.005)
Supplement: Supplement 2 [file NIHMS1930749-supplement-Supplement_2.docx]

**Online Supplementary Table 2. Estimates of the total effect of a standard deviation (SD) increase in measures of pain sensitization on lower extremity function and the corresponding natural direct effects and natural indirect effects mediated by WOMAC knee/hip pain**

|  | WOMAC function (range 0-68) | Chair stand test (number of stands) | 40-meter walking speed (m/s) |
| --- | --- | --- | --- |
| **PPT finger joint** | | | |
| Total effect | **-1.73 (-3.45, -0.02)** | 0.25 (-0.25, 0.74) | 0.03 (-0.01, 0.06) |
| Direct effect | -0.60 (-1.63, 0.43) | 0.14 (-0.33, 0.61) | 0.02 (-0.01, 0.05) |
| Indirect effect | -1.13 (-2.54, 0.28) | 0.10 (-0.04, 0.24) | 0.01 (-0.00, 0.02) |
| **PPT radioulnar joint** | | | |
| Total effect | -1.65 (-3.62, 0.31) | 0.25 (-0.26, 0.76) | 0.03 (-0.01, 0.06) |
| Direct effect | -0.82 (-2.06, 0.41) | 0.17 (-0.30, 0.64) | 0.02 (-0.01, 0.05) |
| Indirect effect | -0.83 (-2.37, 0.71) | 0.08 (-0.07, 0.22) | 0.01 (-0.01, 0.02) |
| **PPT trapezius muscle** | | | |
| Total effect | -1.73 (-3.50, 0.04) | 0.19 (-0.33, 0.72) | 0.04 (-0.00, 0.07) |
| Direct effect | -0.14 (-1.27, 1.00) | 0.05 (-0.47, 0.56) | 0.02 (-0.01, 0.06) |
| Indirect effect | **-1.59 (-2.93, -0.26)** | **0.14 (0.01, 0.28)** | 0.01 (-0.00, 0.02) |
| **PPT tibialis anterior muscle** | | | |
| Total effect | -1.58 (-3.44, 0.28) | 0.12 (-0.51, 0.75) | **0.04 (0.00, 0.08)** |
| Direct effect | -0.49 (-2.01, 1.03) | 0.02 (-0.60, 0.63) | 0.04 (-0.00, 0.07) |
| Indirect effect | -1.09 (-2.53, 0.36) | 0.10 (-0.03, 0.23) | 0.01 (-0.00, 0.02) |
| **Temporal summation** | | | |
| Total effect | 0.93 (-0.97, 2.84) | -0.08 (-0.54, 0.38) | -0.01 (-0.04, 0.03) |
| Direct effect | -0.02 (-0.98, 0.94) | 0.01 (-0.43, 0.44) | 0.00 (-0.03, 0.03) |
| Indirect effect | 0.95 (-0.50, 2.40) | -0.09 (-0.23, 0.06) | -0.01 (-0.02, 0.00) |
| Adjusted for: age, sex, body mass index, ultrasound-detected osteophytes in the knees, hips and feet, education and physical activity.  The effect estimates represent the estimated average increase in physical function outcomes, with corresponding 95% confidence intervals estimated by bootstrapping, presented per sex-specific standard deviation (SD) of the PPT and TS values (SD for PPT at the finger joint in women=1.54 kg/cm^2^ and men=1.78 kg/cm^2^, SD for PPT at the radioulnar joint in women=1.60 kg/cm^2^ and men=2.93 kg/cm^2^, SD for PPT at trapezius in women=1.73 kg/cm^2^ and men=3.72 kg/cm^2^, SD for PPT at tibialis anterior in women=1.90 kg/cm^2^ and men=3.62 kg/cm^2^, SD for temporal summation in women=1.74 and men=1.24).  WOMAC, Western Ontario and McMaster Universities OA Index; m/s, meters per second; PPT, pressure pain threshold | | | |
